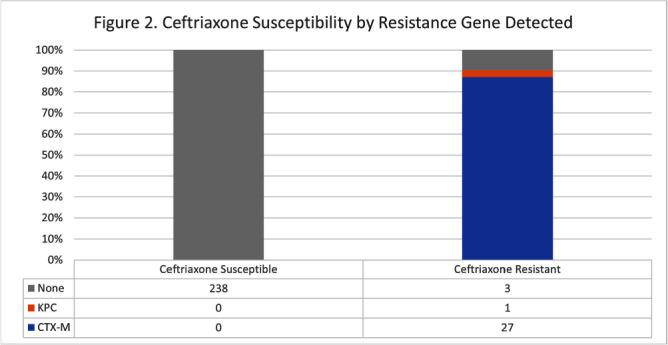# Opportunity for Early De-escalation in Enterobacterales Bacteremia with Rapid Blood Culture Identification Technology

**DOI:** 10.1017/ash.2024.145

**Published:** 2024-09-16

**Authors:** Erin Deja, Jihye Kim, Adam Greenfield, Kimberly Lee, Melissa Godwin, Alexandra Bryson, Sangeeta Sastry, Christopher Doern

**Affiliations:** VCU Health; VCU Medical Center; Vcuhs; Virginia Commonwealth University

## Abstract

**Background:** The BioFire FilmArray Blood Culture Identification 2 (BCID2) Panel is used to identify organisms present in positive blood cultures within hours of detection at Virginia Commonwealth University Health System (VCUHS). BCID2 is also able to detect common resistance mechanisms including CTX-M, the most common extended-spectrum beta-lactamase (ESBL) in the United States, and several carbapenemases. The Antimicrobial Stewardship Program (ASP) at VCUHS established optimal treatment recommendations for each organism identified by BCID2 based on the detection of a resistance mechanism and local resistance patterns. The recommendation for the majority of Enterobacterales without a detected resistance mechanism is ceftriaxone. However, providers are often reluctant to de-escalate antibiotics without confirmed susceptibility testing, as there may be other mechanisms of antibiotic resistance in Gram-negative organisms. The objective of this evaluation was to measure the degree of congruence between BCID2 resistance mechanism detection and susceptibility testing by disk diffusion, and to validate the adequacy of the VCUHS ASP BCID2 treatment recommendations for Enterobacterales bacteremia. **Methods:** Patients with positive Enterobacterales BCID2 results from March 12 to June 19, 2023 were retrospectively identified. Organisms identified by BCID2 that were considered high-risk for clinically significant AmpC production due to an inducible AmpC gene (i.e., K. aerogenes, E. cloacae complex) were excluded. **Results:** A total of 270 results were included. The most commonly identified organism was E. coli (n = 139, 51.5%), followed by K. pneumoniae (n = 74, 27.4%). There were 27 (10%) isolates positive for CTX-M and 1 (0.4%) isolate positive for KPC. All CTX-M isolates were ceftriaxone resistant, and the KPC isolate was meropenem resistant. The remaining 242 isolates were negative for all resistance markers detected by BCID2. Of these, only 3 (1.2%) were resistant to ceftriaxone and notably, 8 (3.3%) were resistant to piperacillin/tazobactam. Overall, BCID2 CTX-M detection was 90% sensitive and 100% specific for predicting ceftriaxone resistance in Enterobacterales. **Conclusion:** CTX-M detection by BCID2 is highly sensitive and specific for predicting ceftriaxone resistance in Enterobacterales. CTX-M negative isolates were more often susceptible to ceftriaxone than to piperacillin/tazobactam, which is commonly used as empiric therapy for Gram-negative organisms at our institution. This highlights an excellent opportunity for safe and effective early de-escalation of antibiotics for treatment of Enterobacterales bacteremia.

**Figure:**
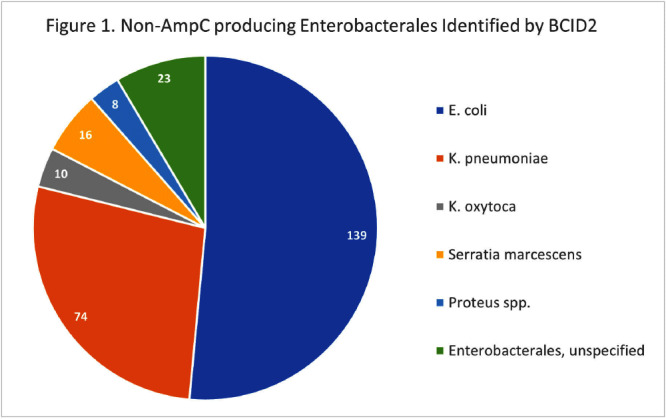

**Figure:**